# Uniportal VATS wedge pulmonary resection

**DOI:** 10.55730/1300-0144.6346

**Published:** 2026-02-12

**Authors:** Kubilay İNAN, Özgür Ömer YILDIZ

**Affiliations:** 1 Department of Thoracic Surgery, Faculty of Medicine, Ankara Yıldırım Beyazıt University, Ankara, Turkiye; 2 Department of Thoracic Surgery, Ankara Yıldırım Beyazıt University Yenimahalle Education and Research Hospital, Ankara, Turkiye

**Keywords:** Interstitial lung disease, pneumothorax, Uniportal VATS, VATS wedge resection

## Abstract

**Background/aim:**

Video-assisted thoracic surgery (VATS) has emerged as a common approach in surgical procedures involving the lung, mediastinum, thoracic wall, and diaphragm. Recent studies have described the performance of diagnostic and therapeutic VATS procedures via a single port, and the technique has become the preferred diagnostic modality for various intrathoracic pathologies and for patients deemed unsuitable for fine-needle biopsy.

**Materials and methods:**

The records of 57 patients who underwent diagnostic and therapeutic uniportal VATS for wedge pulmonary resection between January 2024 and January 2025 were reviewed retrospectively, and details regarding the surgical method, length of hospital stay, complications, and diagnostic accuracy were recorded. The procedures were performed for the diagnosis of interstitial lung disease and for the treatment of undiagnosed pulmonary nodules and primary spontaneous pneumothorax.

**Results:**

Of the total sample, 15.8% (n = 9) were female and 84.2% (n = 48) were male, and the mean age was 39.9 years (range 16–77 years). All 57 patients underwent uniportal thoracoscopic pulmonary wedge resection, with 35 treated for pneumothorax and 22 for other conditions. Those treated for pneumothorax were predominantly younger, with a mean age of 26.6 years ( range 16–77 years), while the mean age of the nonpneumothorax patients was 56 years (28–77 years).

**Conclusion:**

Thoracoscopic surgery is now the preferred method for both patients and surgeons, and the role of VATS in diagnosis and treatment is growing and gaining value. As a minimally invasive procedure, VATS is associated with a comfortable postoperative period for both the patient and surgeon, which supports its role as a safe and feasible approach to wedge pulmonary resections for certain indications.

## 1. Introduction

In recent years, minimally invasive procedures have gained popularity in thoracic surgery. These techniques have gained widespread acceptance and have been improved with experience, with advantages such as reduced surgical trauma, shorter hospital stays, less loss of work, and faster recovery, which shorten the time until patients can start postoperative treatment [1,2]. As surgeons have gained familiarity with the procedure, surgeries that once called for two to three trocars and one utility incision have been replaced by those using one or two trocars and one utility incision. Ultimately, the acceptance of uniportal techniques has been accelerated by the recognition that comparable surgical efficacy can be achieved using only a utility incision.

Uniportal VATS was initially used only for comparatively easier procedures; however, as its benefits have become better understood, doctors are now more willing to apply it to more complicated surgeries, resulting in a growing body of experience in this area. The number of pulmonary nodules found and the number of diagnostic surgical resections carried out have increased as a result of improvements in VATS procedures and the growing use of thoracic tomography [3]. If nodules are detected following routine follow-up inspections, the Fleischner Society recommendations advise surgical excision [4,5].

VATS surgery can be performed for a variety of reasons, including interstitial lung disease (ILD), persistent air leakage, repeated pneumothorax attacks, and bullae, and the procedure allows patients to be assessed and treated in one session. The present study aims to support the optimization of the technique, to advise patient selection, and to increase clinical efficacy through an examination of our uniportal VATS experience, and details the benefits and potential drawbacks identified in global studies.

The primary aim of the present study is to define the quality of VATS by determining its 30-day complication and conversion rates. Secondly, among the indications, the study seeks to determine the feasibility and safety of VATS procedures through a comparison of operative time, chest tube duration, and length of postoperative hospital stay.

## 2. Materials and methods

**Figure 1:**
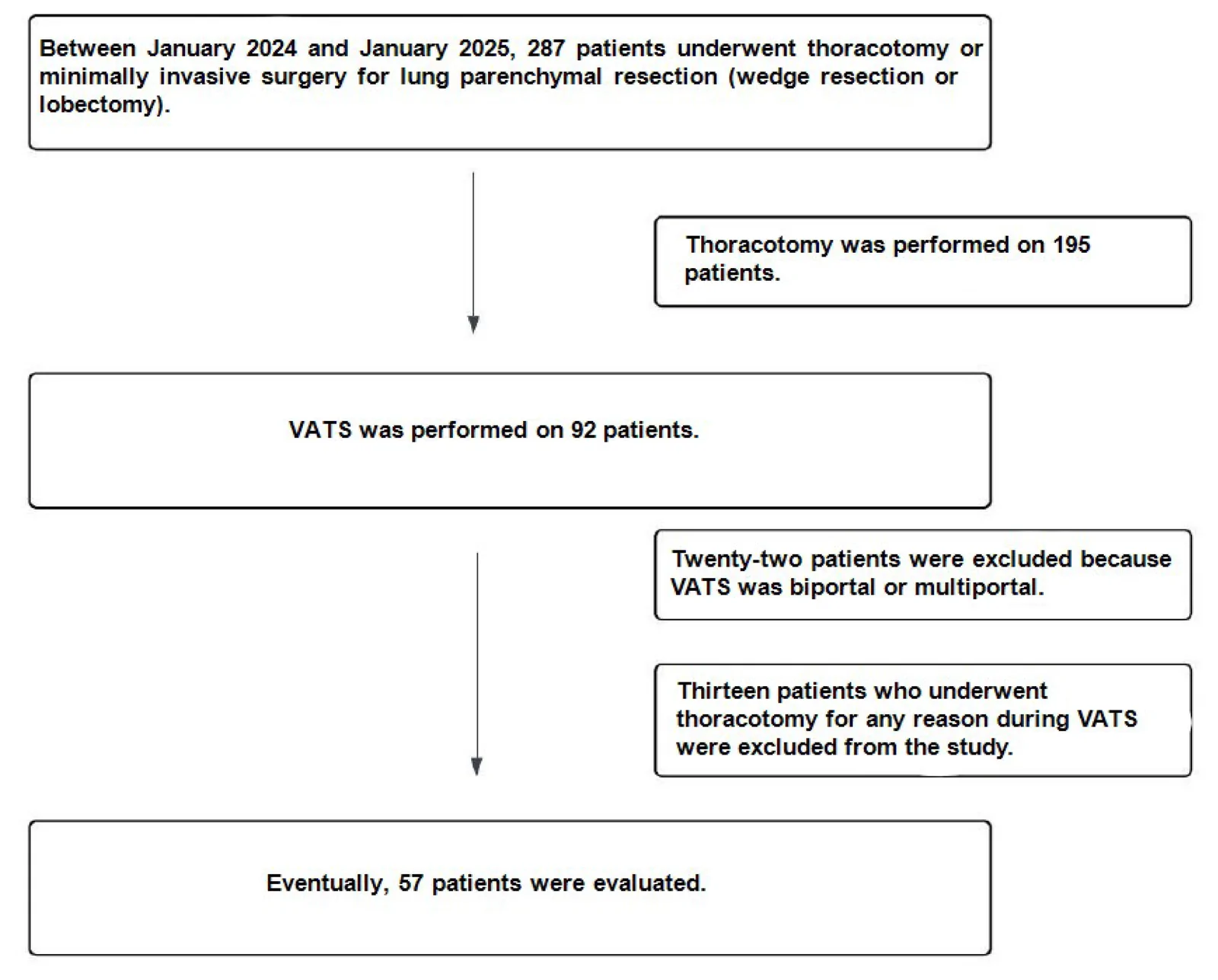
Flowchart of patient selection.

Patients who underwent diagnostic and therapeutic uniportal VATS in our department between January 2024 and January 2025 were reviewed retrospectively. Excluded from the study were individuals undergoing rebiopsy, and those with metastatic and malignant nodules (Figure 1).

The goal in cases of pneumothorax is to completely excise the bullae area without causing air leakage. A frozen section analysis of all wedge resections of pulmonary nodules was conducted, confirming that the nodules were benign. Patients with malignant findings were excluded from the study.

Surgical success was defined as complete removal of the nodule for pulmonary nodules, the establishment of a tissue diagnosis for ILD, and the complete excision of the target area in pneumothorax patients.

Pleural abrasion has been used consistently to prevent pneumothorax recurrence, with the aim being to induce adherence of the visceral pleura to the parietal pleura through the creation of a manual injury.

This study protocol was granted approval by the Ethics Committee of Ankara Yıldırım Beyazıt University Yenimahalle Training and Research Hospital (Ethics Committee No: E-2025-8). Patients were examined based on their age, sex, and comorbidities. Smoking status was classified as never smoked, quit, or active smoker. All patients underwent preoperative thoracic computed tomography, electrocardiography, spirometry (pulmonary function test), complete blood count, and biochemical tests, required to be from within the preceding month.

The success of the surgical procedure was assessed based on 30-day mortality, conversion rate, operation duration, length of stay, and surgical success. Patients were monitored for six months to determine long-term problems, morbidity, and recurrence. Patient follow-up began on day 7 after discharge, with outpatient check-ups every 15 days for the first month, then every 3 months thereafter. The follow-up of all patients is continuing.

Recurrence was defined as the failure to achieve full lung expansion in radiographic examinations performed during the postoperative follow-up period, after full expansion was achieved at discharge.

### 2.1. Surgical technique

**Figure 2:**
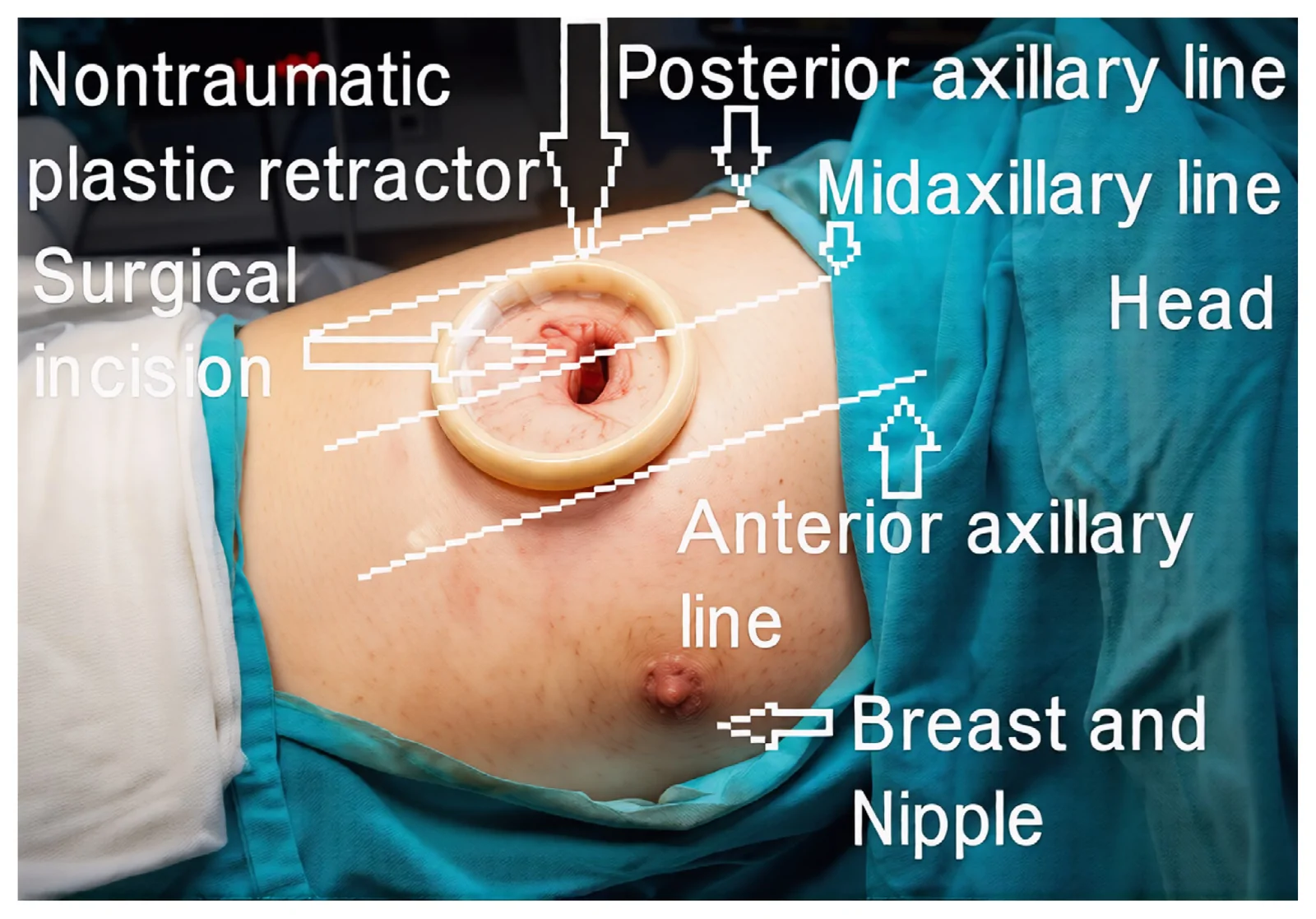
Surgical positioning and incision site.

**Figure 3:**
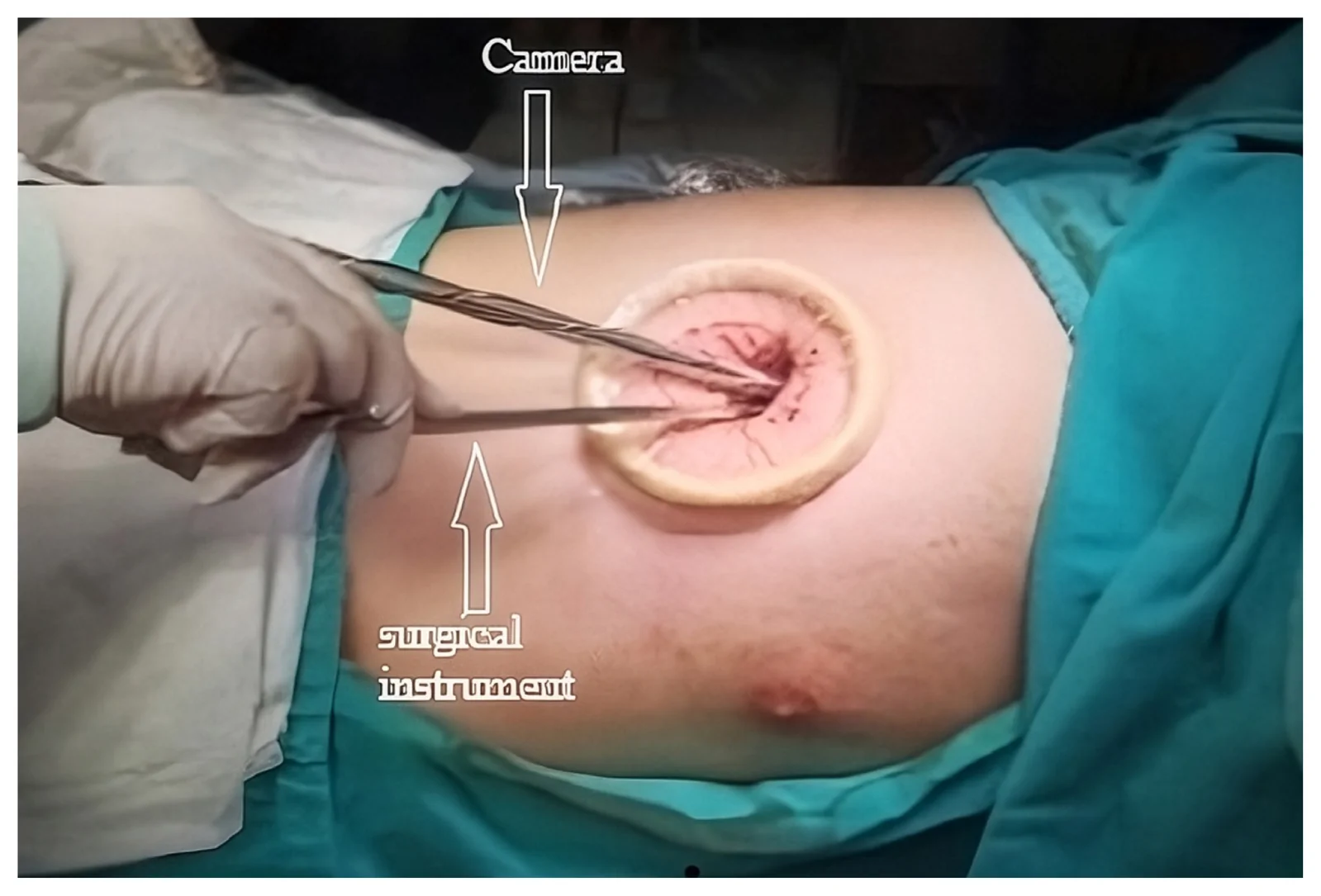
Positioning of surgical instruments at the incision site.

**Figure 4:**
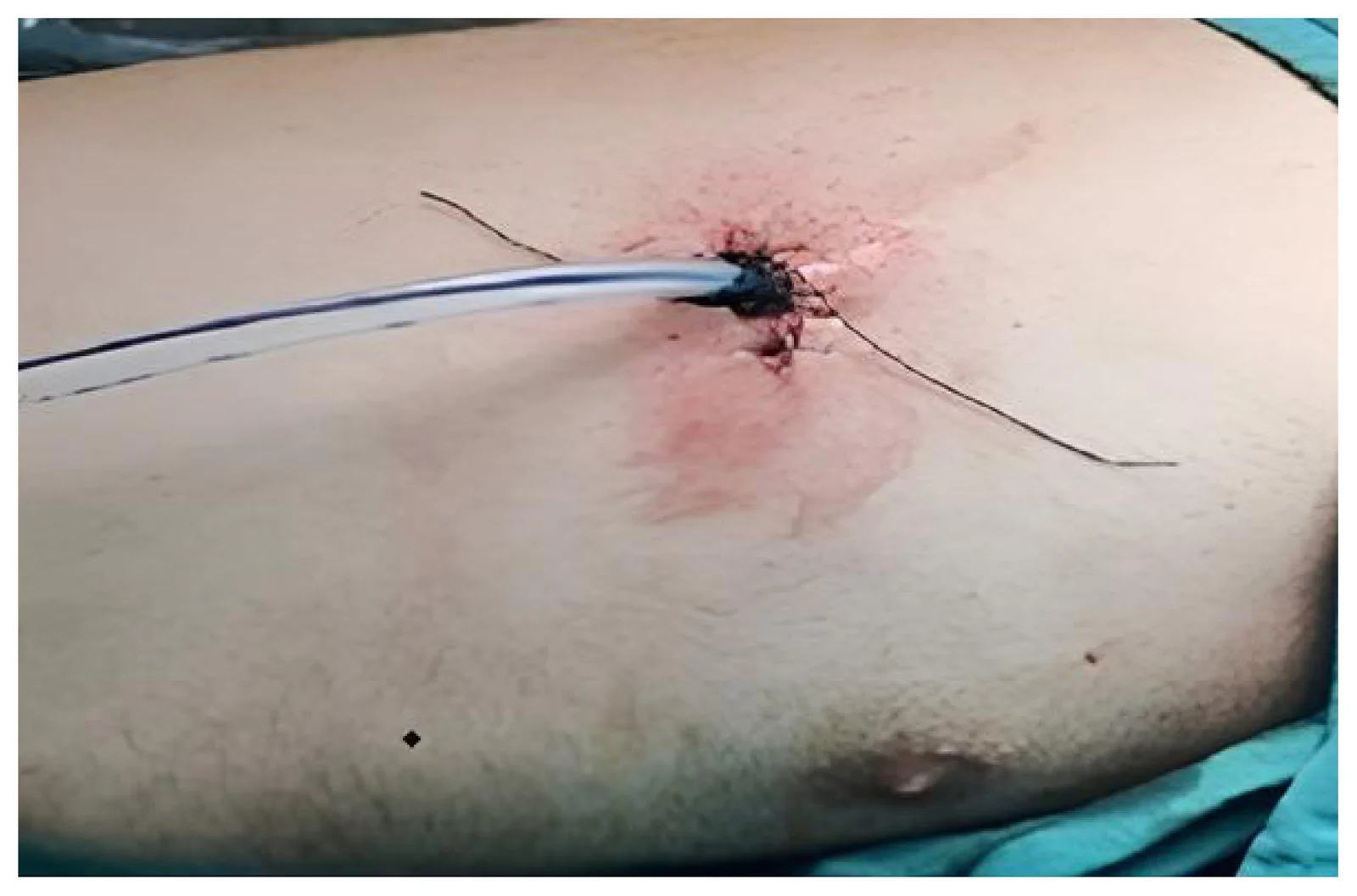
Surgical port site, subsequently used for the chest drain.

Patients were placed in the right or left lateral decubitus position, similar to that used for posterolateral thoracotomy procedures (as shown in Figures 2–4). All procedures were done under general anesthesia with double-lumen intubation. A single 2–4 cm incision was made at the intersection of the fourth or fifth intercostal space and the anterior axillary line, without the use of a rib spreader. A 30-degree camera optic, conventional instruments, a double-articulated endoscopic oval clamp, and a stapler were introduced through the incision.

In patients with extensive ILD, the right lung was particularly selected for the collection of samples from more lobes, and wedge resection of 1–3 diseased lobes was planned intraoperatively. Patients with pneumothorax who underwent VATS wedge resection had either traumatic or spontaneous etiologies.

### 2.2. Postoperative management

A single chest tube was inserted into the same incision (Figure 2) at the end of surgery in all patients to monitor the volume and duration of daily drainage. If the 24-h drainage volume was less than 200 cc and the chest X-ray showed full expansion with no air leak, the drain was removed. Prior to surgery, each patient received a single dose of 1 g of cefazolin for antibiotic prophylaxis. Patients were given nonsteroidal antiinflammatory drugs and opioid analgesics at regular intervals. No routine postoperative antibiotic therapy was administered.

The first 24 h postsurgery were spent in the postoperative primary intensive care unit, after which, monitoring continued in the clinic. Discomfort was evaluated and recorded daily, and the patients were asked to indicate if their discomfort was severe, moderate, or light. The option “none” was added during the control phase.

## 3. Statistical analyses

Statistical analyses were performed using SPSS version 15.0 (SPSS Inc., Chicago, IL, USA). Descriptive statistics were presented as means ± standard deviation for normally distributed variables and as interquartile range (IQR) for nonnormally distributed and ordinal variables. The chi-square test, or Fisher’s exact test (when the chi-square test assumptions do not hold due to low expected cell counts), where appropriate, was used to compare the proportions in different groups. As operation times were not normally distributed, a Kruskal–Wallis test was used for between-group comparisons. A p-value of <0.05 was considered statistically significant.

## 4. Results

Included in the present study were 57 patients who underwent VATS procedures. The procedure was performed for pulmonary nodules in 29.8% (n = 17) of the cases (diagnostic and therapeutic), for pneumothorax in 64.4% (n = 35), and for ILD in 5.8% (n = 5). No patients were identified with preoperative PFT values that could be considered high risk and/or that would prevent surgery. All patients had FEV1 (forced expiratory volume) values of 1.5 L and FEV1/FVC (forced vital capacity) values of ≥80%. The patient demographics and comorbidities are presented in Table 1.

Of the total, 15.8% of the patients were female (n = 9) and 84.2% were male (n = 48). The mean age of the entire sample was 39.9 years (range 16–77 years). The mean age was 26.6 years in patients with pneumothorax, 56.7 years in those with ILD, and 55.6 years in those with pulmonary nodules. The surgical procedure was conducted on the right hemithorax in 29 patients and on the left hemithorax in 28.

The average duration of drainage was 7.2 days, as summarized in Table 2. The pulmonary nodule patients had a mean hospital stays of 8.2 days, compared to 8.9 days for those with ILD, and 6.7 days for those with pneumothorax.

Postoperative drain durations were 2.1 days for pulmonary nodule patients, 2.9 days for ILD patients, and 1.7 days for pneumothorax patients.

The average length of stay for patients during the perioperative and postoperative periods was 9.7 days. Patients with ILD and pulmonary nodules underwent preoperative in-hospital preparations as standard. Patients with pneumothorax were mostly drained preoperatively and were monitored for 1 week for air leakage and expansion defects before surgery. The average length of stay for patients with pneumothorax during the intraoperative and postoperative periods was 9.3 days, while the average postoperative length of stay was 2.1 days. Both the drain durations and lengths of stay were statistically significantly shorter in the pneumothorax group (p < 0.001).

The hospital stay durations of the sample are presented in Table 2.

### 4.1. Perioperative morbidity and mortality

Postoperative pain complaints and complications were the leading factors influencing the duration of hospital stay. In the present study, 25.8% of the patients contracted postoperative pneumonia. The vast majority of patients with pneumonia were older and had ILD (p = 0.003), and comorbidities among these patients were substantial, with a surgical complication rate of 31.5% and a 30-day mortality rate of 0%. Pneumothorax recurred on the operation side in two patients. It was noted that both of these patients continued smoking after surgery. Nasal oxygen was used to monitor patients who developed partial recurrent pneumothorax; no repeat surgeries were required. A smoking cessation program was implemented for the active smokers. Postoperative pain assessment values are presented in Table 3. No patient who underwent uniportal VATS required conversion to thoracotomy.

### 4.2. Operation time

The average surgical time was 43.8 min (34–87 min, sd ± 5.96 min) and was significantly shorter in the pneumothorax group than in the other two groups (p < 0.001).

The proportion of patients with ILD who underwent both lower lobe and upper lobe wedge resection was 55%, and between one and three biopsies were taken in 90% of these cases. These factors, along with the anesthesia-related time loss (switching to double-lung ventilation due to intraoperative saturation decrease, blood pressure increase, and secretion problems, etc.) negatively affected the operation time in this patient group. The surgical times of the sample are presented in Table 4.

## 5. Discussion

The aim of this single-center retrospective study was to improve service delivery quality by evaluating the clinical outcomes of patients with ILD and pulmonary nodules who underwent uniportal VATS wedge resection for pneumothorax indications in our institution.

The latest idiopathic pulmonary fibrosis (IPF) guidelines recommend VATS as a reliable diagnostic approach in cases without a typical interstitial pneumonia (UIP) pattern on chest tomography [6]. The number and location of biopsies may affect the diagnostic efficacy of VATS; therefore, in the present study, the biopsy site was determined based on abnormalities identified from tomography images.

In general, the risk factors were determined to be male sex (p = 0.73), advanced age (p = 0.3), and increased comorbidities (p = 0.04). These risk factors concur with those stated in similar studies [7].

VATS is considered a safe approach to obtaining sufficient lung tissue samples for definitive histological diagnosis, although prolonged air leakage after surgery can lead to more serious consequences than the postoperative benefits, especially in patients with ILD [8,9]. Sigurdsson et al. [9] reported a 30-day mortality rate of 2.7% and a complication rate of 16% in their study, while Kreider et al. [8] reported a morbidity rate of 19% in 68 patients with ILD who underwent VATS. In the present study, the 30-day mortality rate in patients with ILD was 0% and the complication rate was 20%. Similarly, the mortality rate of patients who underwent surgery for pulmonary nodules was 0% and the complication rate was 5.8%. In patients with pneumothorax, mortality was 0% and the complication rate was 5.7%.

There are many factors that influence pain; however, pain is not the focus of the present study, and only the daily levels reported by the participants were assessed and documented, keeping in mind that pain is a subjective condition.

The limitations of this study include its retrospective, single-center design, short follow-up, and small patient sample. As such, the presented results should not be considered a true reflection of the efficacy and safety of VATS in the entire population with ILD. While longer and larger prospective observational studies are needed to validate the results of the study, our results suggest that VATS can guide decisions on whether to administer antifibrotic agents or antiinflammatory agents such as corticosteroids or immunosuppressants.

A 100% diagnostic accuracy rate was observed in the analysis of the postoperative pathology results of patients with ILD and in those with pulmonary nodules.

As reported by Raghu et al., patients with a provisional diagnosis of IPF, including those with histological UIP and those with a possible UIP pattern, have increased mortality after VATS, as described by Hutchinson et al. [10,11]. Consequently, although VATS is not necessary in patients with suspected IPF who have a typical UIP pattern on lung tomography, the procedure can be considered a reliable diagnostic approach in other patients with ILD for the determination of appropriate treatment strategies. In the present study, one patient with suspected ILD who underwent VATS wedge resection was not considered to have UIP radiologically but was identified with UIP pathologically. The patient developed no postoperative complications and was discharged following drain removal.

For pulmonary nodules smaller than 1 cm in diameter detected on CT scan, surgery may be considered if the lesion increases in size or density during follow-up. In the present study, pulmonary vessels were not transected and no segmentectomy was performed during VATS for pulmonary nodules, with procedures limited to wedge resection. This indicates that the surgery was performed within safe limits. Endoscopic staplers were used in all patients in the present study.

Based on the above findings, VATS can be considered a safe, feasible, and economical surgical approach to the precise wedge resection of pulmonary ground-glass nodules with adequate resection margins. Furthermore, this approach can also be considered suitable for patients with pulmonary nodules that cannot be located and biopsied under preoperative CT guidance due to scapular or rib obstruction or proximity to major heart vessels.

Primary spontaneous pneumothorax (PSP) is a common disease caused by the rupture of subpleural blebs. Treatment options include tube thoracostomy with tetracycline, talc, or other pleural irritants; total pleurodesis via thoracotomy; apical parietal pleurectomy via VATS; and apical lung wedge resection as adjunctive or sole therapy [12].

Most cases can be adequately treated with chest tubes; however, recurrence rates are quite high. In a study of patients treated with chest tube drainage [13], the average recurrence rate was approximately 52%. In the present study, patients with pneumothorax, massive air leak, and prolonged air leak who underwent tube thoracostomy underwent surgery. Since patients who underwent tube thoracostomy and who developed recurrence after recovery were excluded from the study, the recurrence rate after tube thoracostomy is not given.

Recurrence rates following VATS or open surgery range from 1% to 14% [14,15]. The low recurrence rate observed with VATS, combined with the short hospital stays, support its feasibility and cost-effectiveness.

In two of the patients who underwent VATS wedge resection for pneumothorax in the present study, partial recurrence developed following the removal of the tube thoracostomy, and treatment with nasal oxygen was administered.

In a German study, chest tubes were removed once thoracic drainage fell below 200 mL—on average 3.4 ± 2.1 days postoperatively—and the mean hospital stay was 8.3 ± 5.3 days [16].

Similarly, chest tubes in the present study were removed once thoracic drainage fell below 200 mL. Preoperative and postoperative drainage durations were calculated separately for the ILD, pulmonary nodule, and pneumothorax patients, with postoperative tube thoracostomy durations of 2.9, 2.1, and 1.7 days, respectively. Similarly, the total and postoperative hospital stay durations of the ILD, pulmonary nodule, and pneumothorax patients were calculated as 10.8, 10.6, and 9.3 days, respectively, and 3.7, 3.2, and 2.1 days, respectively.

## 6. Conclusion

Uniportal VATS is a minimally invasive surgery technique that continues to gain importance for the detection and treatment of lung illnesses. Its utility in diagnosing and treating interstitial and bullous lung illnesses, as well as unexplained nodules, is clearly apparent. The main benefits of the procedure include its minimally invasive nature, avoidance of cosmetic concerns, lower postoperative pain, and shorter hospital stays when compared to open surgery, as well as its cost-effectiveness. We consider uniportal VATS to be a safe and practical treatment that should be considered for other clinical applications.

## Figures and Tables

**Table 1:** Demographic characteristics and comorbidities of patients.

		I.L.D.	Pulmonary nodule	Pneumothorax

**Age (mean year)**		56.7	55.6	26.6

**Sex (number)**	Male	3	15	30
	Female	2	2	5

**Side**	Right	4	10	15
	Left	1	7	20

**Smoking status, n (%)**	Never smoked	2 (3.5%)	2 (3.5%)	4 (7%)
	Quit smoking	2 (3.5%)	4 (7%)	23 (40.4%)
	Active smoker	1 (35%)	11 (19.2%)	8 (14%)

**Comorbidities (number)**	Hypertension	3	9	-
	Diabetes mellitus	4	11	-
	Heart failure	1	2	-
	Renal failure	1	3	-
	COPD	1	4	-
	Asthma	-	3	-

**Table 2:** Drainage and hospitalization durations.

	Drain duration,		Length of stay,	
	postoperative period (days)	Total period (days)	postoperative period (days)	Total period (days)
**ILD**	2.9	8.9	3.7	10.8
**Pulmonary nodule**	2.1	8.2	3.2	10.6
**Pneumothorax**	1.7	6.7	2.1	9.3

**Table 3:** Postoperative pain assessment.

	Pain		
	Postoperative first 24 hours	Postoperative first week	At the 15th day after discharge
**ILD**	Severe	Mild	Low
**Pulmonary nodule**	Severe	Low	Low
**Pneumothorax**	Mild	Low	None

**Table 4:** Durations of surgery.

	Duration of surgery (mean minutes) (minimum–maximum), sd
**ILD**	58 (32–76), sd = ±17 min
**Pulmonary nodule**	61 (23–67), sd = ±10 min
**Pneumothorax**	17 (10–28), sd = 2.3 min

sd: standard deviation

## Data Availability

The datasets used and/or analyzed for the current study are available from the corresponding author upon reasonable request.
